# Testing scientific models using Qualitative Reasoning: Application to cellulose hydrolysis

**DOI:** 10.1038/s41598-017-14281-4

**Published:** 2017-10-26

**Authors:** Kamal Kansou, Caroline Rémond, Gabriel Paës, Estelle Bonnin, Jean Tayeb, Bert Bredeweg

**Affiliations:** 1 INRA, Biopolymères Interactions Assemblages, BP 71267, 44316 Nantes, France; 2 FARE laboratory, INRA, University of Reims Champagne-Ardenne, 51100 Reims, France; 3 Informatics Institute, University of Amsterdam, Science Park 904, 1098 XH Amsterdam, The Netherlands

## Abstract

With the accumulation of scientific information in natural science, even experts can find difficult to keep integrating new piece of information. It is critical to explore modelling solutions able to capture information scattered in publications as a computable representation form. Traditional modelling techniques are important in that regard, but relying on numerical information comes with limitations for integrating results from distinct studies, high-level representations can be more suited. We present an approach to stepwise construct mechanistic explanation from selected scientific papers using the Qualitative Reasoning framework. As a proof of concept, we apply the approach to modelling papers about cellulose hydrolysis mechanism, focusing on the causal explanations for the decreasing of hydrolytic rate. Two explanatory QR models are built to capture classical explanations for the phenomenon. Our results show that none of them provides sufficient explanation for a set of basic experimental observations described in the literature. Combining the two explanations into a third one allowed to get a new and sufficient explanation for the experimental results. In domains where numerical data are scarce and strongly related to the experimental conditions, this approach can aid assessing the conceptual validity of an explanation and support integration of knowledge from different sources.

## Introduction

Keeping up to date in some fields of natural science is getting more and more difficult for the domain specialists since the accumulation of scientific information has been inexorable^1^. For example, searching for “cellulose and hydrolysis and enzyme” in the Web Of Science (WoS) database (01/2017) yields more than 4000 scientific publications since 1995. Even domain experts find it difficult to keep integrating new mechanistic information about cellulose hydrolysis and to envision the consequences on understanding the system dynamics. As the complexity of the systems investigated by researchers grows, so does the difficulty to integrate new pieces of knowledge to the existing explanations. There is a growing need for modelling solutions able to capture the different types and aspects of the scientific knowledge to support the knowledge integration process. Capturing and automatically analysing causal knowledge is fundamental in this regard to provide valuable envisioning of the system behaviours. However, not many tools or methodologies are available to aid domain researchers in doing it.

Mathematical models are a first answer to the problem. Mathematical models embody scientific understanding about the phenomenon under investigation^2^. Regarding our case study, enzymatic hydrolysis of cellulose, kinetics models have long been acknowledged for offering the possibility to represent an aggregated understanding of the mechanism integrating miscellaneous ideas and theories found in the literature^3^. Thus, developing kinetic models has been a significant part of the research on cellulosic substrate enzymatic degradation. Consequently, adding the term “modelling” to the “cellulose and hydrolysis and enzyme” WoS query results in 613 publications and in their review Bansal *et al*.^4^ reported 73 published kinetics models from 1975 to 2009. The published scientific models, in particular differential equations models, represent a fair amount of formalised understanding of the cellulose hydrolysis, expressing distinct, complementary and even competing ideas. It is highly relevant for the domain to analyse those scientific models and to determine which representations are the most promising for further developing an encompassing understanding of the natural system under investigation. As suggested by Zhang and Lynd^5^ this can be done by confronting the outputs of a given model to a range of observations from the domain literature. However, traditional modelling formalisms, such as Ordinary Differential Equation (ODE), have limitations when deployed as an instrument for integration. At some point, the mathematical formulation, the calibration technique, or the need for precise and accurate numerical data (even with relatively simple kinetic models), negatively affect the assessment-readiness of the model structure. In fact, proper instruments are lacking to explicitly capture the scientific understanding (underpinning a kinetic model) into a computational form and based on that generate outputs that can be analysed and compared to a range of observations from other scientific publications.

Our objective is to define a method based on high-level modelling to review scientific models and theories. As case study we focus on publications about cellulose enzymatic degradation. We propose an investigation based on cause-effect reasoning to perform assessment of the structures of kinetic models. Specifically, the *capacity* of the model structure to *explain* observations taken from the literature can be addressed in this way (in addition to the traditional verification and validation techniques, such as goodness of fit, prediction performance, and sensitivity analysis).

Higher level representations, such as Qualitative Reasoning (QR) models cf. ref.^6^, can be deployed to address the above described challenge. QR models use automated cause-effect reasoning as the basis for predicting system behaviour. They do not require numerical information for doing so, and the causal reasoning is mathematically sound cf. ref.^7^. Hence, QR models can map quantitative model structures as well as assemble information from the literature in a *computational cause-effect model*. For this reason, QR models can be used to test the validity of domain theories and scientific models at the qualitative level^8^.

In this paper, we present an approach to stepwise construct a mechanistic explanation from selected papers about cellulose hydrolysis rate slowing-down using the QR framework. Our primary objective is to demonstrate how the QR framework can be used for this. As a proof of concept, we have developed three QR models. Two models are derived from published mechanistic models. The third further enhanced model is derived from experimental observations from the literature and analysis of the simulation results of the other two models. Our paper also introduces methodological issues relevant to creating and assessing such models.

## Declining hydrolysis rate of cellulosic substrates

Cellulose is made of long glucose chains organized into microfibrils that are tightly packed into fibrils, which create into high crystalline or amorphous zones. Complete breakdown of cellulose into glucose by enzymes requires three types of cellulase activities: enzymes attacking glucose chains randomly (endo-glucanases), enzymes starting at chain ends (exo-cellulases so-called cellobiohydrolases) and enzymes cleaving the resulting glucose dimers (cellobiose) into glucose (glucosidases). Kinetics curves typically describe the amount of glucose released over time. The efficiency of the depolymerisation of the solid cellulose chains gradually declines with time. This means that the cellulase activity gets less efficient as the reaction proceeds^3,9,10^. Many studies have investigated the declining hydrolysis rate of cellulosic substrates^4,11^. One active topic of research focuses on processes involving surface enzymes, the exo-acting cellobiohydrolases (CBHs). CBHs are identified as the key contributors of cellulose hydrolysis^12^. They hydrolyse processively cellulose strands from chain ends meaning that the enzyme slides along the cellulose chain during hydrolysis, leading to several catalytic events for each enzyme-substrate complex. However, these enzymes are multi-modular proteins made of a catalytic domain associated to one or more binding domains to the polysaccharide chain. For a long time, it has been presumed that adsorption of non-productive CBH via their carbohydrate binding module could cause steric hindrance on the surface for the processive action, increasing the hydrolysis rate slowing down^13,14^. Other studies explain rate limitation by accumulation of non-productive cellulose enzymes at the surface, until a maximum threshold from which no further adsorption occurs, due to steric hindrance or other unknown reasons^11,15,16^. Valjamae *et al*.^14^ also introduced the surface-erosion model, in which processive hydrolysis causes gradual alteration of the substrate structure, increasing surface heterogeneity. Obstacles at the substrate surface would limit the catalytic activity of the CBHs and foster the accumulation of “stalled” enzyme in a complexed form^17–21^. According to this model, the hydrolysis rate is mainly governed by the desorption rate at which stalled CBHs expulses the strand from their catalytic domains prior to engage in catalytic cycle anew. This model is further supported by observations with real-time atomic force microscopy of “traffic jams” of processive CBH enzymes, stemming from obstructions or obstacles at the surface^22^.

In this paper, we use cellulose hydrolysis rate limitation as our case study, particularly the limiting factors, and focuses on data, explanations and models provided by a set of publications addressing this topic.

## Qualitative Reasoning for mechanism modelling

Qualitative Reasoning (QR) is an area of Artificial Intelligence that focuses on understanding how engineers and scientists reason about physical systems, with the aim to design automated reasoners that achieve expert-level performance cf. ref.^6^. Most QR approaches strive for inferring system behaviour from a description of the physical system structure in a symbolic, human-like manner. A major contribution of QR formalisms is the ability to capture causal reasoning. This includes automatically generating causal accounts of the possible behaviours of a system, in a way that is insightful for humans (experts, scientists, etc.). QR proved useful for different tasks such as supporting decision-making, teaching scientific theories to students, and supporting knowledge discovery through identification of models from data. Traditionally QR has been applied to address engineering or physics problems, but QR models are also proving insightful to model theories^23^, or identify mechanisms, in domains such as sociology, ecology, and biology/bioinformatics where the system structure is ill-defined^6,24^.

**Figure 1:**
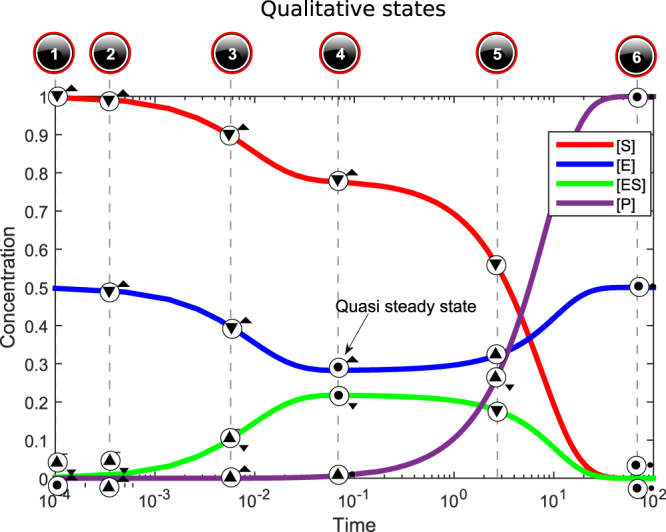
Simulation results for an enzymatic reaction in logarithmic time. The top row shows corresponding qualitative states, produced by simulating a QR model. Value histories of the quantities are placed on top of the simulation curves. Characteristic states are: initial state 1 (substrate starts being complexed with enzyme), state 4 (quasi-steady state), and end-state 6 (substrate conversion complete).

We use Garp3^25^, a workbench for constructing and simulating QR models. To illustrate the use of QR, consider the basic enzymatic reaction: E + S $\rightleftharpoons$ ES → E + P, with E (enzyme), S (substrate), ES (enzyme-substrate complex), and P (product). The Ordinary Differential Equation (ODE) representing this phenomenon computes the derivatives of the E, S, ES and P concentrations. These simulations are well known. Figure 1 shows the kinetic curves (coloured lines), produced with dummy values for the kinetic constants.

The Garp3 approach implements a process-centric view, which emphasizes rates. As such, a Garp3 model of the enzymatic reaction includes four entities (E, S, ES, P) each with a quantity *Concentration*, but also the rates *Ratein* and *Rateout* for, respectively, formation rates (for ES and P) and disappearing rate (for ES). In Garp3, quantities are the model variables characterized by the tuple: <Magnitude, Derivative>. The domain of allowable magnitudes associated with each quantity is called the Quantity Space (QS), i.e. the domain of possible values. *Concentration* [*of E*], *Concentration* [*of S*] and *Concentration* [*of P*] are all assigned the QS: {Zero, Plus, Max}. Zero and Max are two landmarks that correspond to respectively 0% and 100% of the maximum concentration of this entity. Plus represents the interval in-between. The other quantities are given the QS: {Zero, Plus}. All derivatives are given the QS: {▼, Ø, ▲} representing a decreasing, steady, and increasing magnitude, respectively. Garp3 can also compute second-order derivatives over the same quantity space.

Garp3 provides two main primitives for capturing causal dependencies between quantities, direct influence (I + and I−) to model a rate influencing a concentration, and qualitative proportionality (P+ and P−) to model the propagation of changes from one quantity to the next. For example, *Ratein [of A] I* + *Concentration [of A]* means that some rate of A (causing quantity) changes the concentration of *A* (influenced quantity) such as:

$$Ratein[of\,A]I+Concentration[of\,A]\equiv \partial Concentration[of\,A]/\partial t=\ldots +Ratein[of\,A]+\ldots $$

In the case of *Concentration [of A] P* + *Concentration [of B]* it means that an increase in concentration of *A* causes an increase in the concentration of *B*. In fact, P + represents a positive monotonic functional dependency, meaning that there is some increasing monotonic function (*ƒ*) such as:

$$Concentration[of\,A]P+Concentration[of\,B]\equiv Concentration[of\,B]=f(\ldots Concentration[of\,A]\ldots )$$

Garp3 also reasons over inequality relations between quantities. They act as constraints on the simulation space.

**Figure 2:**
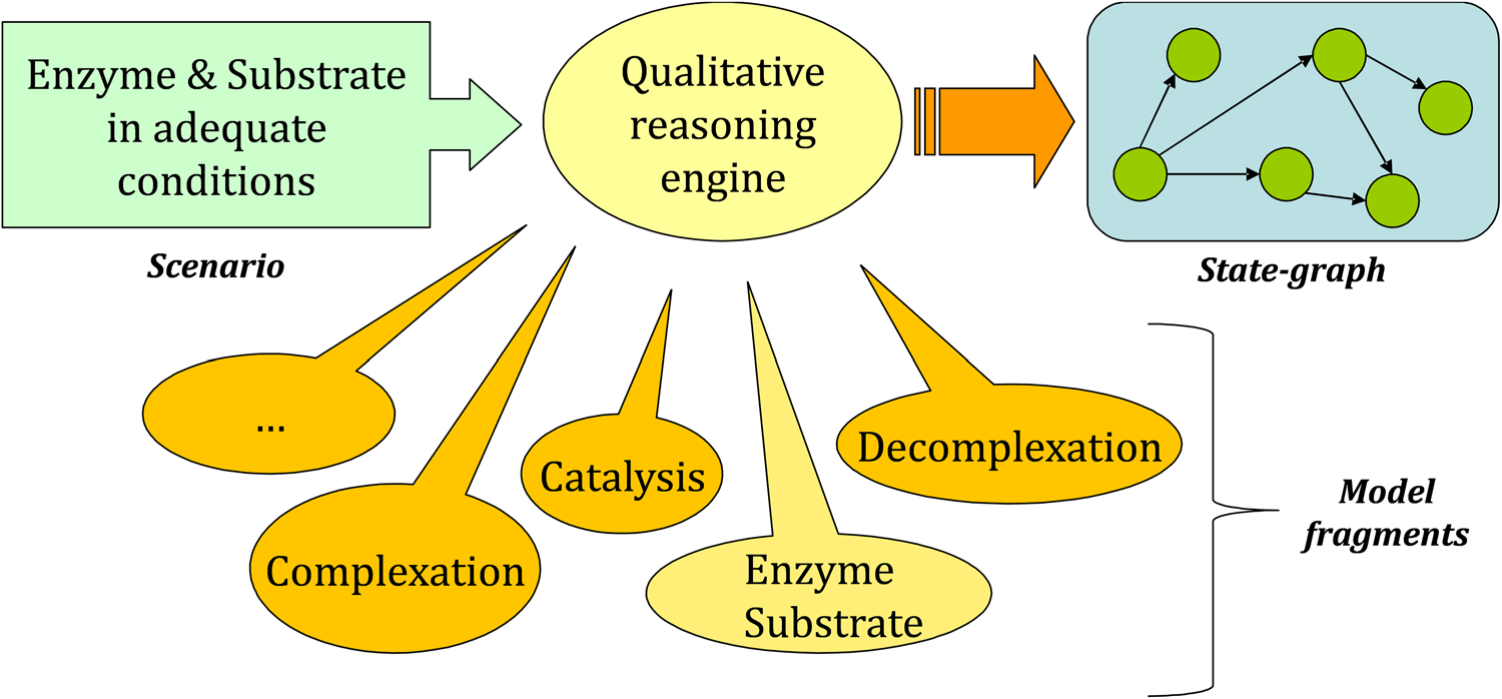
Garp3 architecture. Simulation starts with a scenario. The engine uses a library of model fragments (capturing partial knowledge of processes) to assemble a model fitting the scenario, and then uses that model to incrementally generate the state-graph. In fact, a unique model is assembled for each behaviour state in the state-graph.

The working of the Garp3 software is illustrated in Fig. 2. Simulating a QR model requires specifying an initial situation (scenario). A scenario contains a description of the system including the relevant entities and quantities, and initial values and possibly in/equalities. A scenario may also include the specification of behaviour patterns for quantities that are exogenous to the system, for instance to simulate the consequence of an external perturbation imposed on the system^26^.

**Figure 3:**
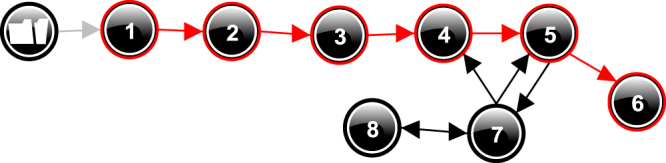
State-graph showing the behaviour states generated for the enzymatic reaction model. Each state (black circle) refers a qualitative distinct behaviour that the system may take on. Arrows refer to state-transitions, e.g. when the system manifests the behaviour represented by state 7 this may change into the behaviours represented by state 4, 5 or 8. Note that the numbers are merely identifiers (they do not refer to state order). The BP [1 → 2 → 3 → 4 → 5 → 6] is shown in red, meaning it is selected by the user.

Back to our example, simulating the qualitative enzymatic reaction model (starting from maximum magnitudes for *Concentration* [*of E*] and *Concentration* [*of S*]) results in a state-graph with 8 states (Fig. 3). Each state represents a qualitatively unique possible behaviour of the system. A Behaviour Path (BP) refers to a succession of states along a complete timeline. Relevant features of the system dynamics can be identified from the state-graph, for instance, evolution towards equilibrium (BP leads to an end state without transition, e.g. [1 → 2 → 3 → 4 → 5 → 6]), but also oscillations (e.g. [4 → 5 → 7 → 4]).

**Figure 4:**
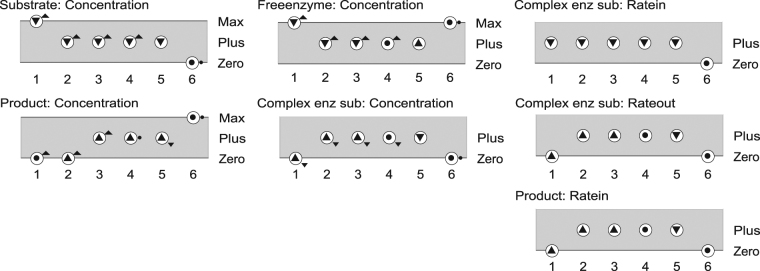
Value history for quantities in the states selected in Fig. 3. “X-axis” refers to the state labels (numbers) in the state-graph. Possible values (as defined by the QS) are listed on the right-hand side of each quantity history. The small circles depicted above each state label denote the current value of a quantity. The tendencies are shown within those circles, one of {▼, Ø, ▲}. For instance, in state 5, *Product Concentration* has current value *Plus*, is *increasing* (∂ = ▲), and *slowing down* (∂′ = ▼, 2^nd^ order tendency shown next to circle).

Figure 4 shows the value history for the quantities in the selected states (Fig. 3). It depicts the evolution of the magnitudes and derivatives over time for these quantities. The value history shows that this particular BP matches the numerical simulation given in Fig. 1. Key qualitative states of the process can be identified, such as state 1 (initial state, substrate starts being complexed with enzyme), state 4 (quasi-steady state), and state 6 (end-state, substrate conversion complete).

States 7 and 8 (Fig. 3) are similar to state 5 but vary for the derivative of *Ratein*[*of ES*] which is steady or increasing (instead of decreasing), respectively. However, these two states provide no additional information about the system dynamic. Such states cover the state space unconstrained regions, and often form cycling behaviours (e.g. [7 → 8 → 7 → 8 → …]). Note that the Garp3 simulation preferences include a “*Fastest path heuristic*” algorithm to filter out this kind of states^25^.

**Figure 5:**
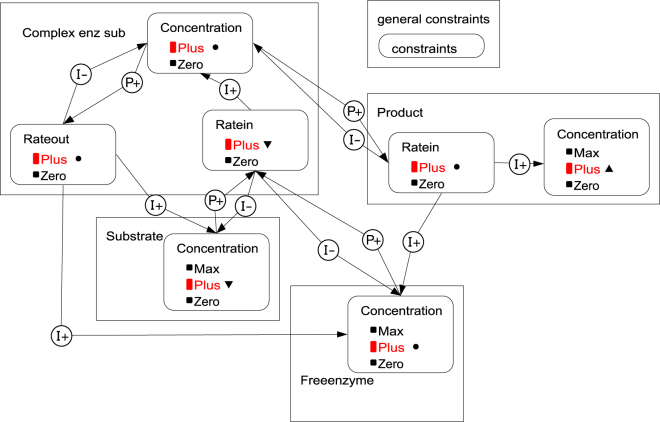
Causal dependencies compiled by Garp3 for state 4 (cf. Fig. 3). The graph provides a causal account for what is depicted by the value history (Fig. 4). Large squares refer to entities (system components). Rounded squares refer to quantities. Current quantity values are coloured red, but can also be identified from the adjacent derivative signs.

Garp3 uses compositional modelling^27^ to build QR models as a set of semi-independent partial models (model fragments) (Fig. 2). Each fragment introduces causal information about the system and its behaviour. Depending on the state of the system (in the model), Garp3 assembles model fragments to interpret that state. As a result, a causal account of the system behaviour is assembled for each state. For example, the chain of causal dependencies at work in state 4 is shown in Fig. 5.

As mentioned before, the representation deploys direct influences (I+ and I−) and proportionalities (P+ and P−). A feedback appears as a loop in the causal diagram. It basically occurs when a chain of causal dependencies relates back to the initial *direct* influence. For instance, *negative* feedbacks exist between the rates and the quantities they influence, such as *Ratein [of P]* and *Concentration [of ES]*: Rate of production of P consumes ES (I−) while being proportional to the concentration of ES (P+). The release of free enzyme along with the rate of production is modelled through a larger *positive* feedback loop: *Ratein [of P]* I + to *Concentration [of E]*, *Concentration* [*of E]* P + to *Ratein [of ES]*, *Ratein [of ES]* I + to *Concentration [of ES]*, and *Concentration [of ES]* P + to *Ratein [of P]*.

## Materials and Method

Now let us focus on the method we propose to review scientific models and theories using automated cause-effect reasoning. Section 4.1 summarizes the basic steps, while Section 4.2 discusses the notion of Target Behaviour; a key concept within the method. Finally, Section 4.3 briefly reports on experiments carried in the context of our modelling effort. Performing additional experiments is not an inherent feature of the proposed method. In our case the experiment was considered relevant to better understand enzymatic hydrolysis, particularly the restart phenomenon.

### Literature integration method

**Figure 6:**
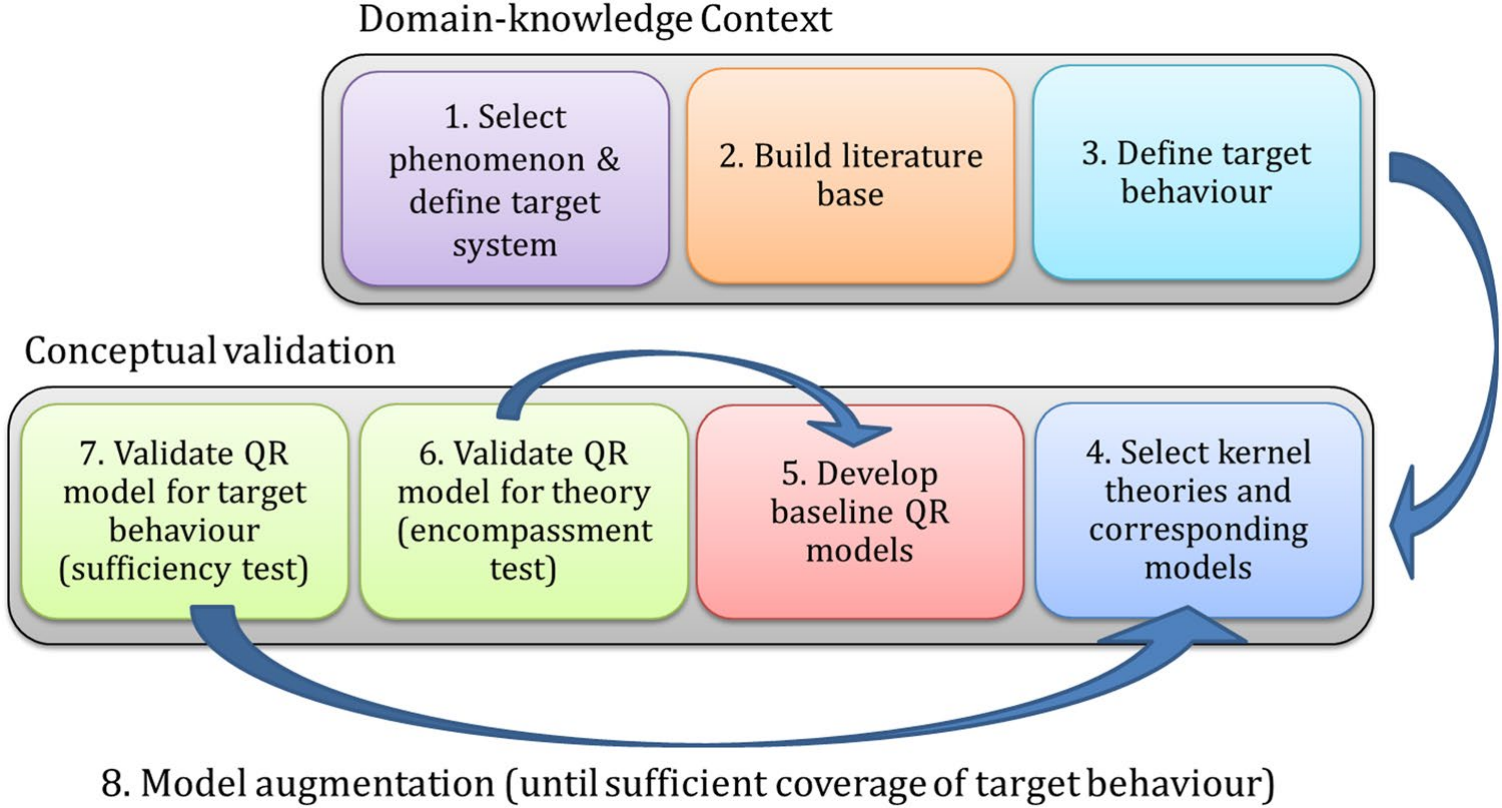
Major steps of the method for reviewing scientific models and theories using Qualitative Reasoning. The method includes a wide analysis of the domain-knowledge context about the phenomenon under investigation, whose main outcome is the target behaviour, and specific analysis of published kernel theories through conceptual validation of corresponding Qualitative Reasoning models.

This method has the following basic steps (illustrated in Fig. 6).

1. Select phenomenon and define target system
2. Build literature-base
3. Define target behaviour
4. Select kernel theories and corresponding models
5. Develop baseline QR models
6. Validate QR model for theory (encompassment test)
7. Validate QR model for target behaviour (sufficiency test)
8. Model augmentation (until sufficient coverage of target behaviour)

The idea is to systematically follow the procedure until a model is found that sufficiently covers (and thereby explains) the target behaviour.

The **first** step is to select a phenomenon that will be investigated and for which an explanation is sought. In practice, this step requires the most basic expression of the phenomenon, with an account of the minimal set of sufficient and necessary components and conditions to observe it. This composes the *Target system*, namely the smallest physical system that exhibits the phenomenon to be explained. Only major entities of the system are selected at this point. The mechanism explaining the system behaviour remains largely unknown and is actually investigated during the following stages. The **second** step is to accumulate a reasonable set of publications that can act as the literature-base. The literature-base is the main resource of knowledge and data to describe the phenomenon and the associated theories. Help from expert scientists investigating the phenomenon is critical at this stage to more easily find and select these publications, identifying trustworthy and enlightening observations as well as important domain theories. The **third** step is to define the *target behaviour*. The purpose of this step is to gather information (as found in the literature-base) on how the target system behaves. It defines the target behaviour for which a mechanistic explanation is sought. The **fourth** step is to select promising theories, possibly competing, about the phenomenon, which can act as a starting point for the development of the QR models. In the ideal case, quantitative (mechanistic) models have been developed to illustrate the theories and can be mapped into qualitative models cf. ref.^28^. The source may come from an item in the literature-base, but may also come from other places, such as expert’s understanding. Using this background material, the **fifth** step is to develop the first QR models. Each model acts as a *baseline* and should reflect the background material adequately. We refer to validating this adequacy as the *encompassment test* and it is executed in the **sixth** step. In fact, steps 5 and 6 alternate until a version of the model is reached that meets the encompassment test. Step 6 is thus completed when a baseline QR model is established that conveys the underlying theory correctly. The **seventh** step validates whether the model also matches the target behaviour (referred to as the *sufficiency test*). This test is similar to idea of an *event validity* test^8^. If the model passes this test, it by definition provides a sufficient explanation for the target behaviour, and thus for the phenomenon as such. In that case, the goal of the procedure is accomplished and further work lies outside the procedure. For instance, the resulting QR model can be used as an instrument to state hypothesis and steer further investigation of the real system if needed cf. ref.^29^.

If in step 7, the model does not pass the sufficiency test, then this model provides an insufficient explanation of the target behaviour. The modeller now may want to test concurrent domain theories (step 4) or augment the model so as to pass the sufficiency test (step 8). In the **eighth** step, a ‘supplementary promising piece of explanation’ is selected from the literature-base (accumulated in step 4) and the QR model is augmented accordingly to include it. Note that, with the QR model this is typically achieved by adding a selection of model-fragments that together (with the already created model) refine the behaviour of the original QR model (this way of working is known as compositional modelling). Each time an augmented version of the ‘latest’ QR model is established, the procedure moves to step 7 to perform the sufficiency test.

### Target behaviour

In our method, it is fundamental to capture the salient characteristics of observational data and to determine whether the QR model reproduces it (and be independent of a given experimental context as much as possible). To this end we introduce the concept of *Target Behaviour*.

A Target Behaviour (TB) is a qualitative abstraction of one or more observations of actual behaviours exhibited by the real (target) system, whose mechanism is unknown and investigated by domain scientists. A TB acts as a filter to assess a candidate qualitative model, by identifying Behaviour Paths (BP) in the state-graph of this model (cf. Fig. 3) that are consistent with it. A TB captures distinct relevant features of the phenomenon as Target States ordered in time for which the candidate model needs to provide an explanation. A Target State describes the target system for a given time period (t), through a set of quantities with known magnitudes (*α*) and/or derivatives (*β*). *α* and *β* can be point or interval values. The candidate qualitative model must include the quantities of the Target State to have a chance of satisfying it. Moreover, *α* and *β* need to map onto Qualitative States (QSs) of the candidate qualitative model (note, derivatives only use QS: {▼, Ø, ▲}). If *α* or *β* can take on any value, this is noted as “?”.

In agreement with the QR formalism, a TB represents sequence of changing magnitudes and/or derivatives of at least one quantity in successive time intervals (referred to as states). Contrary to a BP, a TB does not need to cover a complete timeline, that is, from an initial state to an end state. A TB is defined as a finite sequence of m Target States (TS), strictly ordered in time such as:

$${\rm{TB}}={{\rm{TS}}}_{0}\to \cdots \to {{\rm{TS}}}_{{\rm{i}}}\to \cdots \to {{\rm{TS}}}_{{\rm{m}}}$$

TS_i_: Target State in the i^th^ position. The successor relation ( → ) indicates simply that the next target state occurs some time later. Two successive Target States must be distinct and thus refer to different *α* and/or *β* for at least one quantity. It is desirable but not mandatory that a TB covers a continuous time-period to avoid false positives.

Consider the curves in Fig. 1. We can identify characteristic periods and define them as states, such as (*i*) initial-state of the reaction, (*ii*) intermediate state where [ES] is at a peak, and (*iii*) end-state. These are characteristic states of the system under investigation. Then a possible TB could describe magnitudes and derivatives for the ES and P concentrations at the three moments (t_0_ < t_1_ < t_2_), as shown in Table 1.

**Table 1:** TB of the qualitative features the curves in Fig. 1. ‘?’ can be one of {▼, Ø, ▲}.

Time index	Concentration [ES]	Concentration [P]
t_0_ (initial state)	<Zero, ?>	<Zero, ?>
t_1_ (intermediate state)	<Plus, Ø>	<Plus, ▲>
t_2_ (end state)	<Zero, Ø>	<Plus, Ø>

Now consider the example QR model of the enzymatic reaction (Section 3). It produces the BP [1→2→3→4→5→6] (Figs 1 and 5), which is consistent with Table 1: state 1 of the BP matches the initial-state of the TB, state 4 matches the intermediate (quasi-steady) state, and state 6 the end-state. In fact, all BPs containing these 3 states in the right order are consistent with Table 1. QR outputs being explanatory, this illustrates how a QR model can automatize the production of a sufficient explanation for a TB from a description of the system structure.

### Additional experiments

In addition to observations found in the literature, concrete experiments of enzymatic hydrolysis on cellulosic substrate (Avicel) with a cocktail of cellulases were carried out. Hydrolysis of 1% (w/v) Avicel (Avicel PH-101, Sigma-Aldrich) was performed with a cellulase cocktail from *T. reesei* TR3012 (strain from IFPEN, France) with a loading of 10 mg proteins/g Avicel. Reactions were carried out in 50 mM citrate phosphate buffer (pH 4.8) with chloramphenicol (100 ppm) in a thermostatically controlled system Tornado Radleys® at 45 °C under agitation at 150 rpm. The straightforward enzymatic hydrolysis (REF) was replicated three times. For restart experiments, a second enzyme loading (10 mg/g) was performed after either 1 h (Res1) or after 24 h (Res24), both were replicated four times. Glucose release was quantified by a glucose oxidase assay with an Analox GL6 glucose analyzer (Imlab, Lille France) as described elsewhere^30^.

### Data availability statement

The model and simulation results generated during and/or analysed during the current study are included in this published article (and its Supplementary Information and Supplementary materials files).

## Results

### Defining target behaviour (Step 2 and 3)

Following the above presented method (Section 4.1), we want to establish an explanation for the processes limiting enzymatic hydrolysis of cellulose (Section 2). To compose the TB (step 3), a review of publications pertaining to the cellulose hydrolysis rate decline was performed (step 2). We strived for selecting publications addressing the most basic conditions, involving common cellulosic substrates with common hydrolytic cellulase, typically exo-active cellulase Cel7A from *Trichoderma reesei* (Tr) fungus, with processive activity (enzyme complexed on a cellulose strand chops it up step-by-step as cellobiose units). The goal was to extract observations caused by basic processes that take place regardless of the cellulosic substrate nature or the enzymatic cocktail complexity.

**Figure 7:**
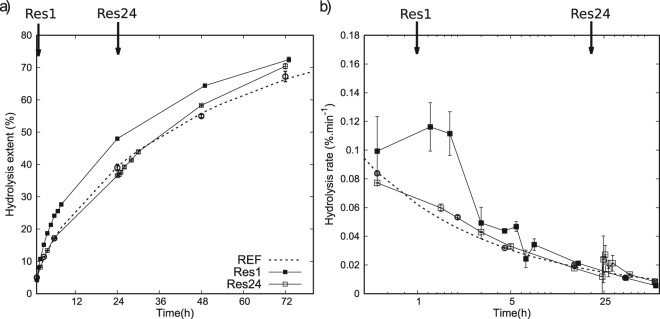
Hydrolysis of Avicel by cellulase cocktail from *T. reesei*. *Left*: Three hydrolysis curves of cellulose Avicel at 1% with 10 mg/g enzymes: without addition of fresh enzyme (REF), with addition of 10 mg/g enzymes at 1 h (Res1), and addition at 24 h (Res24) (arrows indicate the additions). *Right*: The corresponding instantaneous rates showing an exponential decrease over time with a marked restart of hydrolysis for Res1. Dash lines represent arbitrary fits to guide the eye on REF data-points. Error bars indicate standard errors.

*Hydrolysis rate decline:* this rate is related to the absolute quantity of bound enzymes as well as the specific rate per adsorbed enzyme^9^. The rate of hydrolysis of cellulosic substrate decreases rapidly and continuously as the reaction proceeds. The phenomenon extends over different time-scales^31,32^. From onset on to the steady state it may take from 1 minute, up to 1 hour^9,19,20^, up to a few days, depending on the substrate and enzymatic cocktail^33–35^. However, the hydrolysis rate decreases exponentially immediately after an initial burst of catalytic activity and then continues to decrease at a much slower pace^19,20,31^. Figure 7 depicts hydrolysis rates and gives an idea of the time-scales at which the kinetic can be analysed. It shows the initial burst of hydrolysis, followed by a sharp decrease during the first hours of the reaction.

*Restart experiments:* Among the experiments used to investigate the declining hydrolysis rate, “perturbations” of the system are realised by adding fresh enzymes during the course of the reaction. These “restart experiments” provide information about the system state, in particular about the state of the enzymatic component^9,17^. Restart experiments may or may not include substrate washing to remove cellulase from the substrate surface prior to being exposed to a second dose of enzyme^36^. It has been observed that adding fresh enzymes a short time after the reaction initialisation causes a clear restart of the hydrolysis^19,20^. If the addition of fresh enzymes comes after a few hours, a weaker restart is observed, except when the cellulose surface is cleaned beforehand^35,36^.

In house experiments of enzymatic hydrolysis on Avicel with a commercial cocktail of enzymes were performed to confirm observations found in the literature (Section 4.3). Results are given in Fig. 7, which shows a reference hydrolysis with a single dose of enzyme at the start (REF), the addition of a second dose of enzyme after one hour (Res1) or after 24 hours (Res24). The results show the initial burst of hydrolysis, followed by a sharp decrease during the first hours. The experiments show a marked increase of the glucose production rate right after the addition of a second dose of enzyme at one hour (Res1), referred to as ‘restart’. One can see that it is limited in time, after 24 h the production rate of Res1 is similar to that of the reference (REF). The impact of a second dose of enzyme at 24 hours (Res24) is less instantaneous, but seems to spread over a longer period as eventually the hydrolysis extent catches up with the hydrolysis extent of Res 1 at 72 hours.

Based on this information, we propose three TBs (TB1, TB2 and TB2′) to capture prominent aspects of the experimental observations (Tables 2–4). TB1 concerns the hydrolysis decline rate and more specifically the initial burst of hydrolysis. The long-term decline is postponed to future work. TB2 depicts the restart behaviour as the conversion of free enzyme into catalytic active enzyme, such that the catalytic rate increases as long as the free enzyme quantity is increasing. TB2′ also concerns restart, but differently from TB2, there are processes limiting and possibly interrupting the restart phenomenon prematurely, so that the increase in free enzyme may not result in an increase of the catalytic rate comparable to TB2.

**Table 2:** Declining hydrolysis rate after an initial burst of hydrolytic activity (TB1).

Time index	Free enzyme	Catalytic rate
t_0_	<Max, ?>	<0, ▲>
t_1_	<{Zero, Plus}, ?>	<Plus, ▲>
t_2_	<{Zero, Plus}, ?>	<Plus, Ø>
t_3_	<{Zero, Plus}, ?>	<Plus, ▼>

**Table 3:** Second dose of enzyme brings about a hydrolysis restart (TB2).

Time index	Free enzyme	Catalytic rate
t_0_	<Plus, ▲>	<Plus, Ø>
t_1_	<Plus, ▲>	<Plus, ▲>
t_2_	<Plus, Ø>	<Plus, {Ø, ▲}>

**Table 4:** Limited hydrolysis restart due to extra processes (TB2′).

Time index	Free enzyme	Catalytic rate
t_0_	<Plus, ▲>	<Plus, Ø>
t_1_	<Plus, ▲>	<Plus, ▲>
t_2_	<Plus, ▲>	<Plus, Ø>
t_3_	<Plus, Ø>	<Plus, ?>

### Qualitative model development (step 4 and 5)

We selected two classical theories about cellulose hydrolysis limitation. The first one focuses on limitation of access to the cellulose surface due to enzyme adsorption. The second model envisages the presence of obstacles at the cellulose surface that cause the stalling of processive enzymes. These theories propose alternative explanations. Both have been implemented as mathematical models and presented in publications. They are our main resource for designing the baseline QR models.

### Select kernel theory (step 4)

The first theory is the surface-coverage limitation explanation. The cellulose surface crowding by enzyme has been suspected for a long time and tends to be confirmed by recent studies^11,16^. Maurer *et al*.^16^ propose a reaction-kinetic model based on modified Langmuir-Michaelis-Menten equations to simulate how surface finiteness can limit further adsorption of enzyme. The system accounts for three processes:

1. Reversible adsorption on the surface
2. Reversible formation of surface enzyme-substrate complex
3. Hydrolysis of substrate generating a product

### The principle of the model

is described by the following reaction:

1 $${E}_{b}+OS\,\begin{array}{c}\mathop{\to }\limits^{{k}_{A}}\\ \mathop{\leftarrow }\limits_{{k}_{D}}\end{array}\,{E}_{s}\,+\,S\,\begin{array}{c}\mathop{\to }\limits^{{k}_{1}}\\ \mathop{\leftarrow }\limits_{{k}_{-1}}\end{array}\,E{S}_{S}\,\mathop{\to }\limits^{{k}_{2}}\,P$$

The corresponding mass balance relates the surface concentration of open adsorption site (*OS*) and the bulk enzyme concentration (*E*_*b*_) to the production rate (*∂P/dt*) via the surface concentration of adsorbed cellulase in an uncomplexed (*E*_*s*_) and complexed (*ES*_*s*_) form. *S* represents the surface cellulose chain available for complexation. It is assumed constant in the model. *OS* is controlled by the following conservation relation:

2 $${{\rm{\Gamma }}}_{max}=OS+{E}_{s}+E{S}_{s}$$

where ${{\rm{\Gamma }}}_{max}$ is the maximum surface concentration of the substrate.

The second domain theory supposes the presence of obstacles on the cellulose surface that limit the processive action of the exo-active enzymes as put forward in Jalak and Valjamae^18^, also implemented as reaction-kinetic model in Prastegaard *et al*.^19^ and in Cruys-Bagger *et al*.^20^. The kinetic model implements the stalling of processive enzyme when it reaches a surface obstacle during the catalytic process. Principle of the model reaction is as follows^20^:

3 $$\begin{array}{l}E+{C}_{m}\mathop{\to }\limits^{{k}_{on}}E{C}_{m}\mathop{\to }\limits^{{k}_{cat}}E{C}_{m-1}\cdots \mathop{\to }\limits^{{k}_{cat}}E{C}_{m-n}\\ \quad \quad \,{k}_{off}\downarrow \quad \,{k}_{off}\downarrow \quad \quad \quad {k}_{off}\downarrow \\ \quad \quad \quad E+{C}_{m}\quad E+{C}_{m-1}\,\,E+{C}_{m-n}\end{array}$$

*E* represents a processive cellulase (cellobiohydrolase). The enzyme adsorbs on cellulose surface and complexes with a cellulose strand following a rate constant, *k*_*on*_. Contrary to the model of reaction (1), here the adsorption and the complexation steps are lumped together. *C* represents the cellulose strand concentration; a cellulose strand is composed of *m* cellobiose units. Enzyme hydrolyses in a processive manner following *k*_*cat*_, to release cellobiose. It is assumed that on average *n* units of cellobiose are released by the enzyme before it gets stalled by some obstacles at the cellulose surface as *EC*_*m-n*_ complex. Complexed enzyme dissociates at a rate constant, *k*_*off*_. Little is known about the origin and nature of the surface obstacle. *n* is assumed constant by Cruys-Bagger *et al*., and *C* is in high excess and its influence can be neglected.

### Develop baseline QR model (step 5)

**Figure 8:**
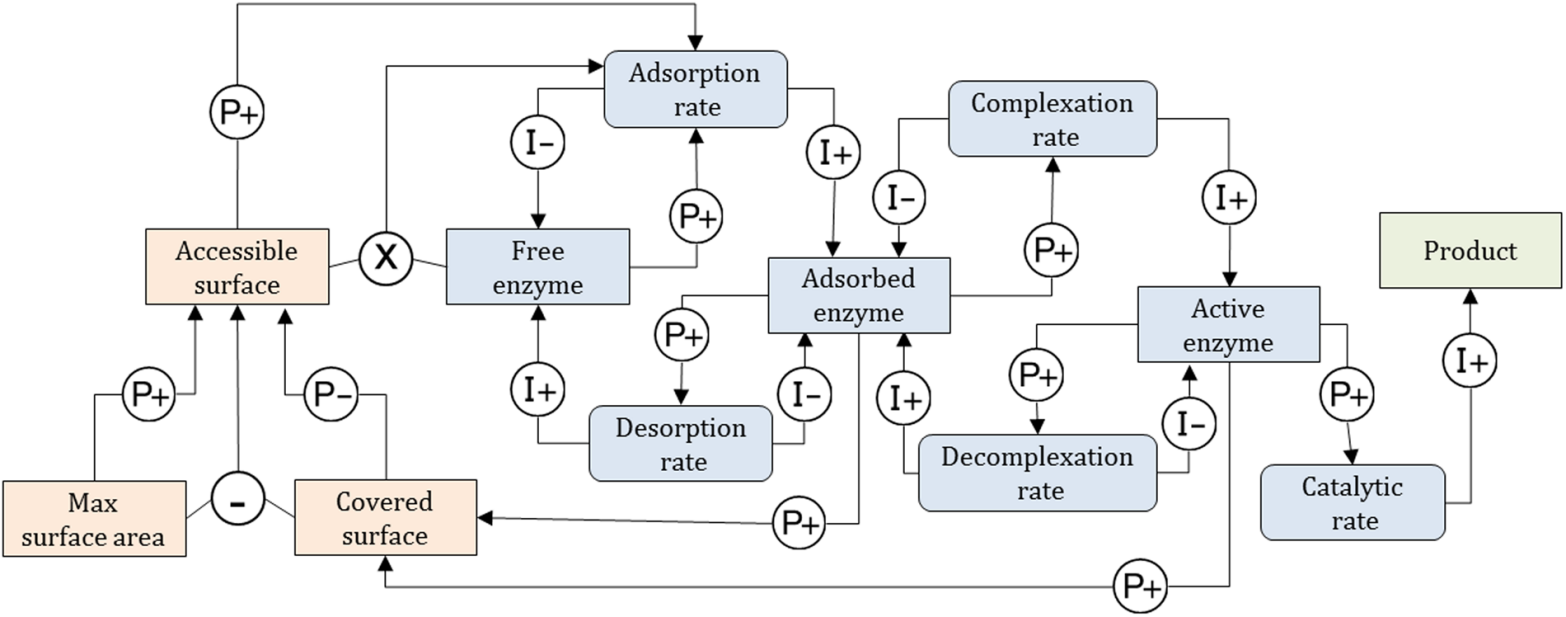
Qualitative Reasoning Model M1 for surface limitation. Square boxes represent *concentrations*. Rounded boxes represent *rates*. Causal linkages and are labelled I+/− (Influences) and P+/− (Proportionalities). Influences relate rates to concentrations. Proportionalities propagate information between concentrations, and from concentrations to rates. Algebraic relations can be used in Garp3 through qualitative algebra. Operators are represented by ⊕, ⊖ and ⊗. The mapping with the numerical model (1) and (2) is given in Table 5. Orange denotes substrate-related quantities, blue-grey is used for enzyme related quantities, and green denotes product related quantities.

**Figure 9:**
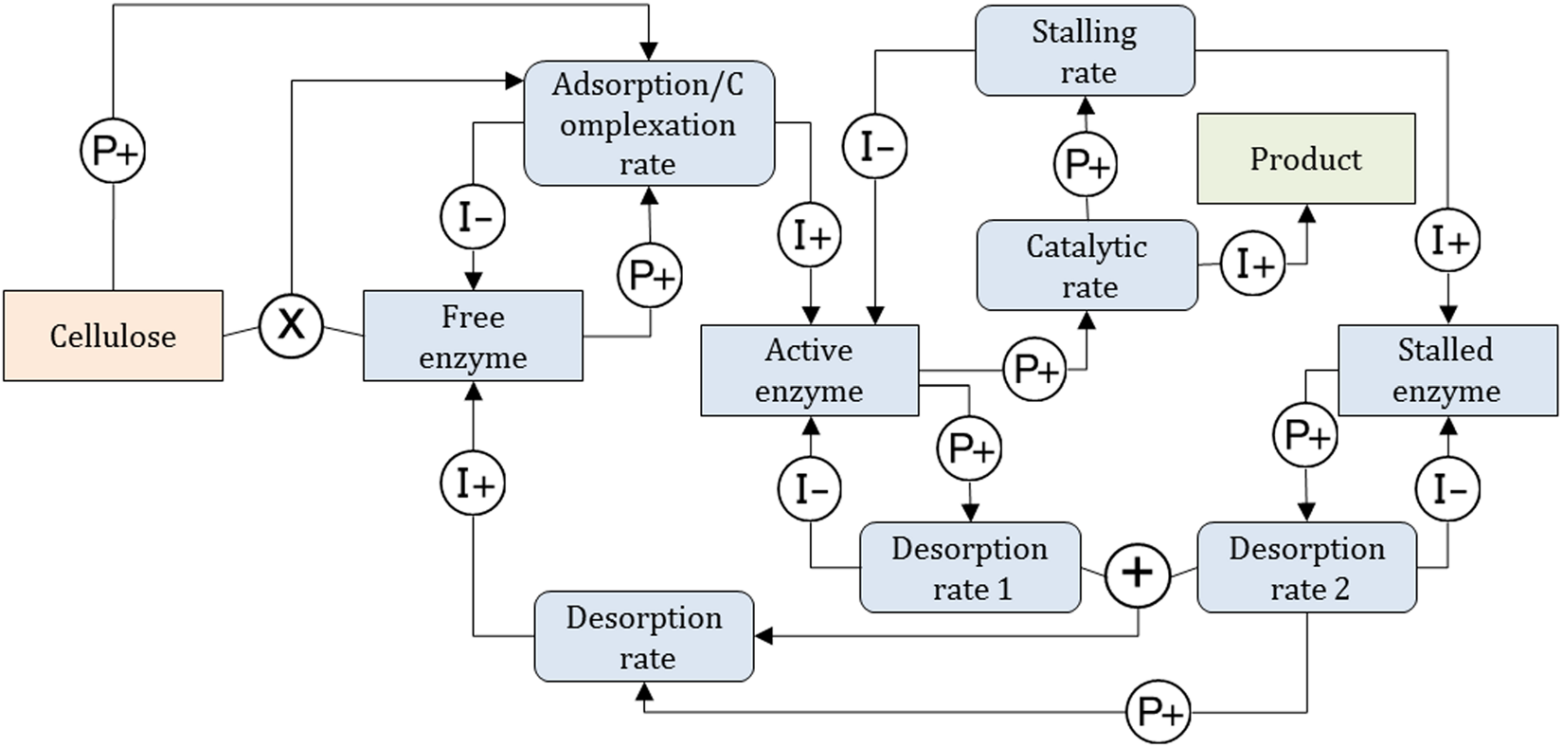
Qualitative Reasoning Model M2 for stalling of enzyme. Relationship with the numerical model (Eq. 3) is given in Table 5. Orange denotes substrate-related quantities, blue-grey is used for enzyme related quantities, and green denotes product related quantities.

We developed two baseline QR models to map the structure of the two kinetic models presented above. Correspondences between quantities of the source models and those of their QR counterparts are reported in Table 5. To present the models structure we adopted a diagrammatic representation describing the causal linkages between the quantities (Figs 8 and 9).

**Table 5:** Variables used in the source models and their quantity counterparts in the QR models.

Source model notation		QR models	Description
reaction 1,2	reaction 3	Quantities	
*E*_*b*_	*E*	*Free enzyme: concentration*	Free enzyme in solution
*E*_*s*_	—	*Adsorbed enzyme: concentration*	Enzyme non-productively adsorbed on cellulose
*ES*_*s*_	*EC*_*m-I*_, *i∈{0,n-1}*	*Active enzyme: concentration*	Enzyme productively bound to a cellulose (of *m* cellobiose units in Eq. 3)
*Γ*_*max*_	—	*Max Surface Area: concentration*	Maximum surface adsorption sites
*OS*	—	*Accessible surface: concentration*	Open surface adsorption sites
*P*	—	*Product: concentration*	Product from hydrolysis
—	*C*_*m-j*_, *j∈{0,n}*	*Cellulose: concentration*	Cellulose string of *m-j* cellobiose units
—	*EC*_*m-n*_	*Stalled enzyme: concentration*	Enzyme productively bound blocked at *n* cellobiose units

QR model M1 (surface limitation) is depicted in Fig. 8. *Free enzyme* first adsorbs onto *Accessible surface* to form *Adsorbed enzyme*. *Adsorbed enzyme* can form *Active enzyme* by complexation with a cellulose strand. *Active enzyme* can either degrade the cellulose at *Catalytic rate*, or go back to the *Adsorbed enzyme* form at *Decomplexation rate*. The model implements surface limitation through negative feedback loops: *Accessible surface* quantity is proportional (P+) to the *Adsorption rate*, which influences (I+) the amount of *Adsorbed enzyme*, and the amount of *Active enzyme* via the *Complexation rate*. Both *Adsorbed enzyme* and *Active enzyme* are proportional (P+) to *Covered surface*. Finally, *Covered surface* is inversely proportional (P−) to *Accessible surface*.

Note that kinetic constants (${{\rm{k}}}_{{\rm{A}}},{{\rm{k}}}_{{\rm{D}}},\ldots )$ are parameters that typically convey quantitative information. They do not appear in purely QR models. Here they are embedded in the corresponding rates. For instance, *Adsorption rate* (Fig. 8) does not appear as such in the mathematical model of reaction (1) but stands for ${{\rm{k}}}_{{\rm{A}}}[{{\rm{E}}}_{{\rm{b}}}][{\rm{OS}}]$. This is captured in Garp3 as the result of the product between *Free enzyme* and *Accessible surface* and two positive proportionalities (P+) (Fig. 8).

**Figure 10:**
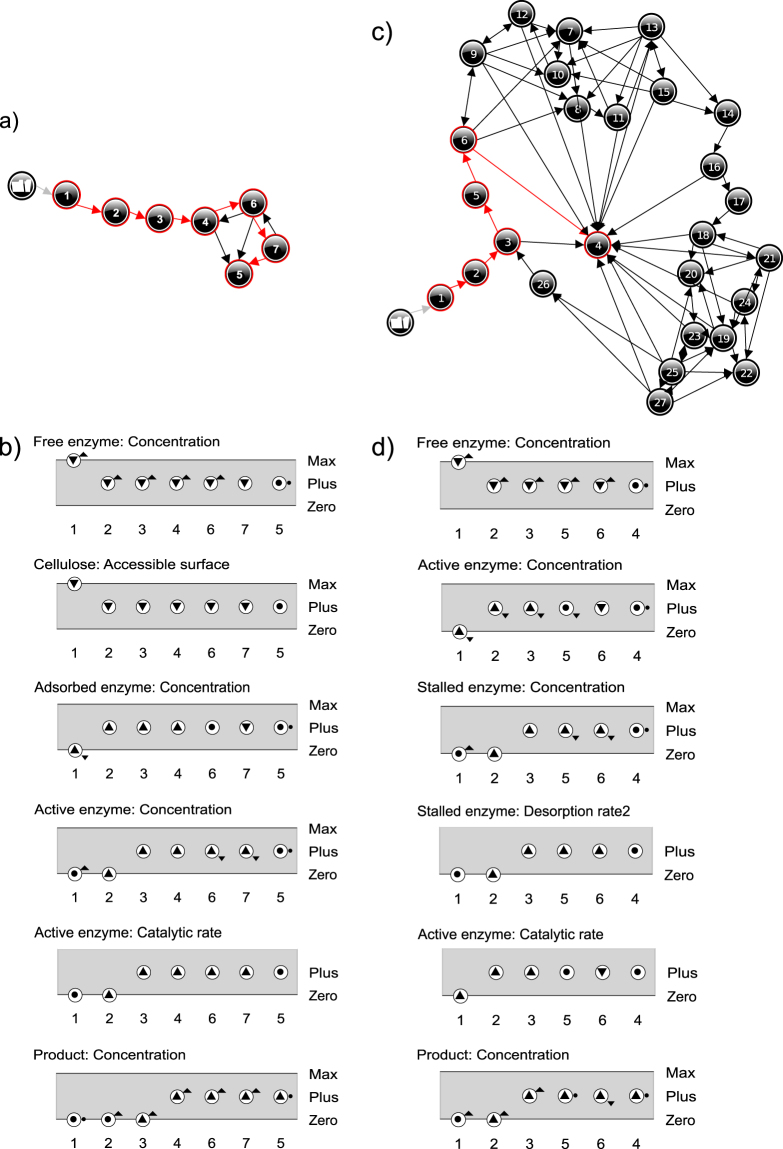
Simulation results for M1 and M2. State-graph of model M1 (**a**). Value histories of selected quantities for model M1 depicting the behaviour path [1→2→3→4→6→7→5] (**b**). State-graph of model M2 (**c**). Value histories of selected quantities for model M2 depicting the behaviour path [1→2→3→5→6→4] (**d**). See Figs 8 and 9 for an overview of the relations between the quantities of M1 and M2, respectively.

Simulation results of M1 are shown in Fig. 10a,b. The state-graph has seven states ordered linearly. It has one steady end-state (state 5) where the catalytic rate is constant and the product quantity increases linearly. For all possible BPs (BP [1→2→3→4→6→7→5] is selected in Fig. 10a) the *Catalytic rate* increases up to a steady state. The simulation envisions a straightforward conversion of *Free enzyme* first into *Adsorbed enzyme* and then into *Active enzyme*. *Adsorbed enzyme* increases until it reaches a peak. Thus, the BP of Fig. 10a depicts a kinetics where *Adsorbed enzyme* decreases (state 7) prior to stabilizing (state 5). The surface limitation, reflected by the decreasing *Accessible surface*, is caused by the accumulation of *Adsorbed enzyme* and *Active enzyme*. With M1, *Accessible surface* diminishes along with the amount of *Free enzyme*, meaning that both are susceptible to explain the reaction rate limitation.

QR model M2 (stalling on obstacles) is shown in Fig. 9. This model produces *Active enzyme* from a global *Adsorption/complexation rate* of *Free enzyme*. *Active enzyme* degrades the cellulose strands processively at *Catalytic rate*. Next, it can either desorb (*Desorption rate1*) or get stalled if it meets an obstacle at *Stalling rate* and becomes *Stalled enzyme*. The *Desorption rates* (*Desorption rate1* + *Desorption rate2*) refill the amount of *Free enzyme* fuelling the turn-over. In model M2, hydrolysis is a single step process performed by all *Active enzyme* and not a summation of hydrolytic acts occurring along the cellulose strands as in reaction (3). At the qualitative level, it would make the system and the resulting explanation needlessly complicated. Here again, the original model of the reaction (3) makes use of kinetic constants (k_on_, k_off_, k_cat_) that are embedded in the corresponding rates, in particular *Desorption rate1* stands for k_off_[EC_m-j_] with j ∈ {0,n-1}, *Desorption rate2* for k_off_[EC_m-n_] and *Stalling rate* for k_cat_[EC_m−n+1_]. *Stalling rate* is positively related to the *Catalytic rate* therefore the relation between the *Catalytic rate* and the *Stalling rate* is modelled using a proportional dependence (P+).

Simulation results of the implemented QR models are shown in Fig. 10c,d. M1 and M2 produce very different state-graphs. The range of possible behaviours is larger for M2 than for M1, despite a comparable number of quantities. The state-graph of Model M2 has 27 states with a characteristic water lily leaf shape (Fig. 10c) and a unique end-state at the centre (state 4). Similar to state 1 in the simulation results of M1, state 4 is an equilibrium state in which all quantities of the system are steady, except for the concentration of *Product*, which increases linearly at a constant *Catalytic rate*. After a common starting branch (states 1→2→3) the system either: (*i*) goes directly to state 4, hence BP [1→2→3→4], or (*ii*) initiates oscillations before reaching state 4, or (*iii*) oscillates without reaching the end state. The system’s behaviour presents some analogy with that of a damped oscillator moving towards a steady state. Steady quantities in state 4 (Fig. 10d) allows deriving equalities between rates from analysis of the influence relations of model M2 (Fig. 9):

**Table:** 

State 4 – Given	Derived equalities between rates
*δ*(*Free enzyme*)/*δt* = 0	*Adsorption/Complexation rate* = *Desorption rate Adsorption/Complexation rate* = *Desorption rate1* + *Desorption rate2*
*δ*(*Active enzyme*)/*δt* = 0	*Adsorption/Complexation rate* = *Desorption rate1* + *Stalling rate*
*δ*(*Stalled enzyme*)/*δt* = 0	*Stalling rate* = *Desorption rate2*

The initial branch [1→2→3→…] depicts the constitution of a stock of *Stalled enzyme*, therefore inevitable with this model. There is a constant amount of *Stalled enzyme* at the equilibrium state.

### Validate QR model for theory (encompassment test)

To investigate the encompassment of M1 for the interpretation by Maurer *et al*.^16^ simulation curves have been produced from implementation of the ODE model as described in the publication (see Supplementary Figs S1 and S2). The longest BP (7 states) produced by M1 maps exactly onto the numerical simulation (see Supplementary Fig. S2). It depicts the burst and then the decline of *Adsorbed enzyme*, while *Active enzyme* increases up to maximum level at which it stabilizes. >From Fig. 8 it is easy to trace back the limitation of the amount of *Active enzyme* to the depletion of *Accessible surface* and/or *Free enzyme*. As a matter of fact, deleting the model fragment in the QR model associated with surface limitation (concerns the details shows in the orange boxes in Fig. 8), does not change the state-graph for this scenario.

**Figure 11:**
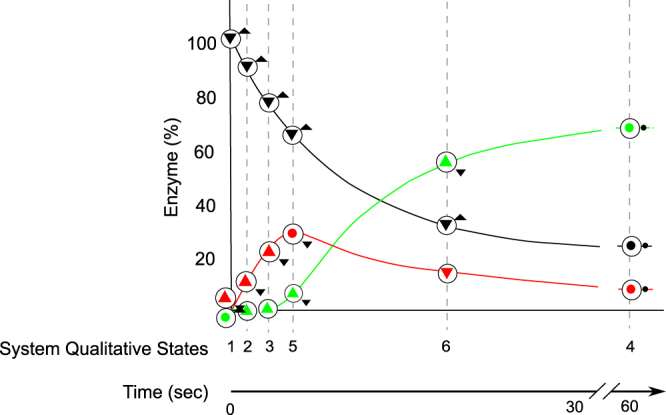
Enzyme evolution in M2 *vs* simulation data from the source publication. The evolution of the percentage of enzyme in each state for the BP [1→2→3→5→6→4] in x-axis, is placed on top on actual simulation curves from Cruys-Bagger *et al*.^20^. Red is Active enzyme, black is Free enzyme and green is Stalled enzyme.

The encompassment for M2 regarding Cruys-Bagger *et al*.^20^ is depicted in Fig. 11. The BP [1→2→3→5→6→4] matches the simulation curves provided in that publication. From state 2 to state 6, *Stalled enzyme* increases then *Stalling rate* > *Desorption rate 2* (from influence in Fig. 9) until it levels out in state 4 (*Stalling rate* = *Desorption rate 2)*. The imbalance in favour of *Stalling rate vs Desorption rate2* creates a bottleneck effect via accumulation of stalled enzyme. This slows-down the hydrolytic activity and affects the overall efficiency of the hydrolysis reaction

Interestingly, the shortest BP of the state-graph ([1→2→3→4]) also matches another experimental curves provided in Cruys-Bagger *et al*.^20^, obtained with the lowest substrate concentration (0.25 g/L of amorphous cellulose with 50 nM of cellobiohydrolase TrCel7A). Here, the hydrolysis rate levels out close to its maximum value so that there is no noticeable burst. Absence of burst means that the *Adsorption/Complexation rate* is limitative compared to the other rates (Desorption and Stalling rates).

### Validate QR model for target behaviour (sufficiency test)

**Figure 12:**
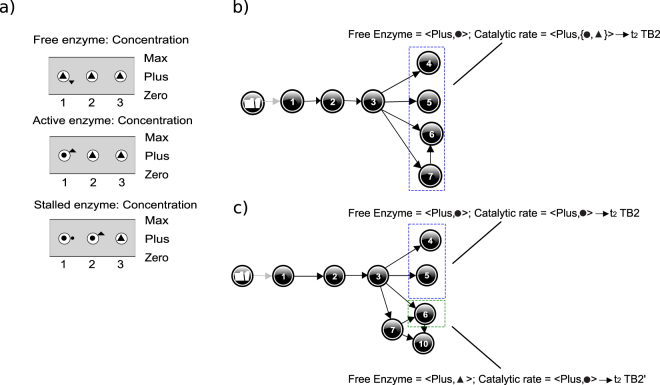
Partial simulation results for the Restart scenario for models M2 and M3. It includes the value history of the 3 first states for M2 and M3 (**a**), the first steps of M2 simulation in agreement with TB2 (**b**), and the first steps of M3 simulation in agreement with TB2 and TB2′ (**c**).

Results of the sufficiency test are shown in Table 6. M1 and M2 provide incomplete explanation for one of the three TBs. Particularly, M1 produces no BP with a decline of the hydrolysis rate, TB1 (Fig. 10b). Indeed, following *Active enzyme* evolution, the *Catalytic rate* increases then stabilizes, which does not satisfy TB1, t_3_ (Table 2). M2 provides an explanation for the decline of the *Catalytic rate* (directly proportional to the concentration of *Active enzyme*) in agreement with TB1 (Figs 10d and 11). TB2 and TB2′ are assessed in the QR models through a dedicated scenario that mimics the addition of *Free enzyme* in a system at the equilibrium, with a forced increase of *Free enzyme*. M1 produces BPs in line with TB2 (see Supplementary Fig. S3): addition of *Free enzyme* generates a restart of the hydrolysis process, and with TB2′ (see Supplementary Fig. S3): the reduction of *Accessible surface* due to the accumulation of *Adsorbed* enzyme and *Active enzyme*s can counteract the restart due to more *Free enzyme*. M2 compliance to TB2 is detailed below. First steps of this simulation are shown in Fig. 12a,b.

**Table 6:** Results of the sufficiency test.

Model	TB1	TB2	TB2′
M1	—	X	X
M2	X	X	—

Addition of *Free enzyme* increases the adsorption of enzyme on the cellulose and, necessarily, brings about the increase of *Active enzyme* ([1→2→3] in Fig. 12a,b). This is consistent with TB2, t_0_ and t_1_ (Table 3). In Fig. 12b, all branches from state 3 onwards go through a stabilization of *Free enzyme* (*Free enzyme* = <Plus, ●>, in states: 4, 5, 6, 7) and *Catalytic rate* = <Plus, ●>, in states 4, 5 or *Catalytic rate* = <Plus, ▲> in states 6, 7. These configurations comply with t_2_ of TB2 (Table 3). The simulation depicts a behaviour where the second dose of *Free enzyme* is completely transformed into *Active enzyme*, and causes a burst of hydrolysis anew. This behaviour matches well with the real kinetics observed for short-duration hydrolyses as reported in Praestgaard *et al*.^19^ and in Cruys-Bagger *et al*.^20^. Regarding TB2′ (Table 4), no BP from M2 complies with the t_2_ stage: *Free enzyme* = <Plus, ▲> ∧ *Catalytic rate* = <Plus, Ø>. It is clear from Fig. 12b that with M2 the stabilization of the quantity of *Free enzyme* is a necessary condition for the stabilization and then for the decline of the *Catalytic rate*. M2 cannot account for a disrupted restart. Further analysis of the compliance to TB2′ for M2 (and later for the augmented model M3) is provided in section 5.5.

### Model augmentation (step 8 and 7)

Sufficiency test results and further consultation of domain publications can be used to focus the augmentation of the baseline QR models towards complete coverage of the TB set.

Additional theory. Looking at the sufficiency test results (Table 6), it would be desirable to somehow combine M1 and M2 into a third model, M3, to cover all the three TBs. A first task is to determine which parts of M1 and M2 to re-use in the new model. M1 matches TB2′ in the sufficiency test due to surface limitation counterbalancing addition of fresh enzyme effect. Therefore, it is desirable to have surface limitation in M3. Regarding M2, the stalling of enzyme can explain the declining hydrolysis-rate before the establishment of the steady-state kinetics, a behaviour that cannot be obtained with M1. So M2 should be the base for M3.

To our knowledge there is no existing theory or models to augment M2 with surface limitation processes. To guide the conceptualization of how the augmented model could work, we considered the following assertions from publications:

1. Bansal *et al*.^37^ measured a sharp decline of the fraction of productively adsorbed enzymes with time, more pronounced for higher enzyme loadings. This is consistent with processes impacting the complexation.
2. Given the larger size of the cellulase compared to the cellulose strand width, it would not be surprising if enzyme getting immobilized at the surface, irreversibly or not, caused steric hindrance blocking other cellulase acting on adjacent cellulose strands^36^.

### Augment baseline QR model

**Figure 13:**
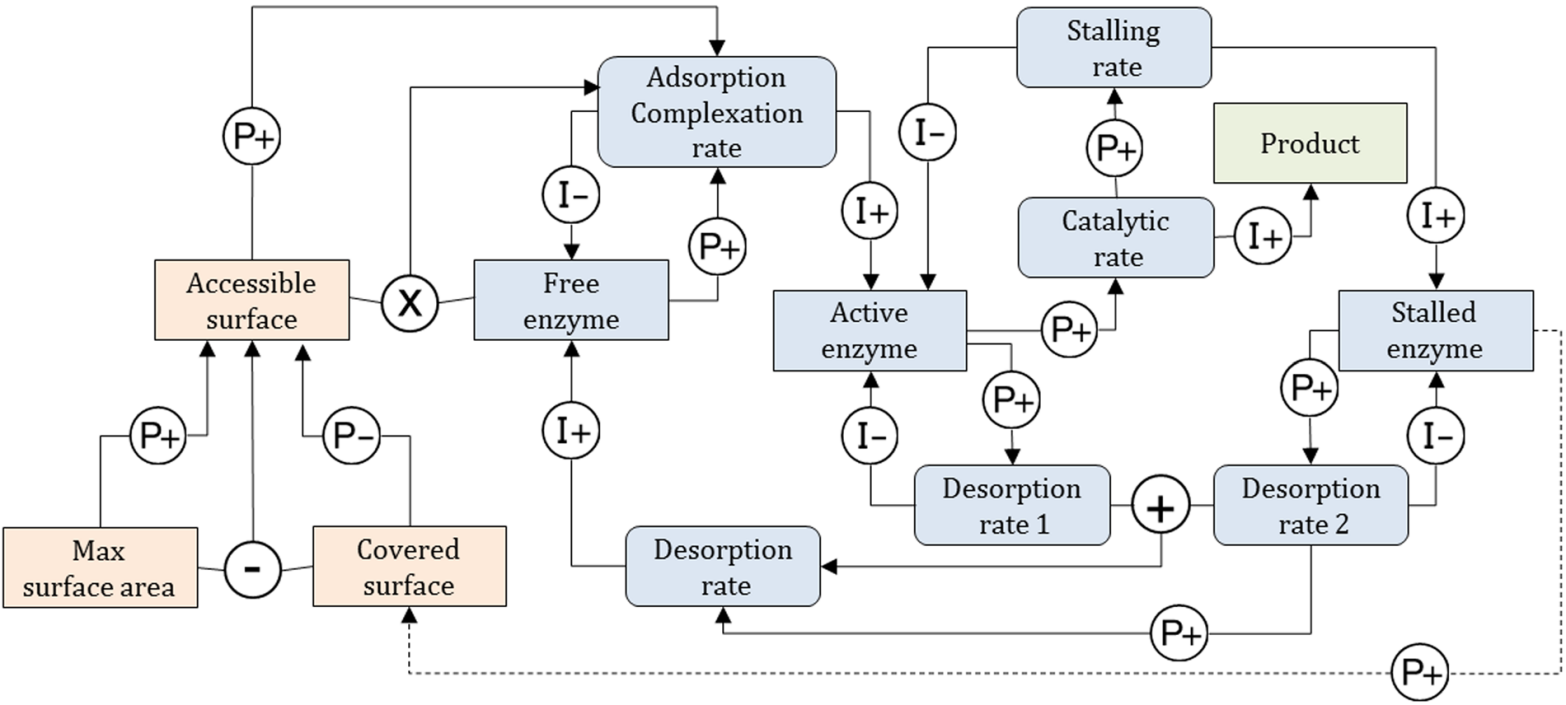
Qualitative Reasoning Model M3. Relationship with the numerical model (Eq. 3) is similar to M2 (Fig. 9). However, here *Cellulose* is replaced by a model fragment that computes *Accessible surface* from *Max surface area* and *Covered surface*. Orange denotes substrate-related quantities, blue-grey is used for enzyme related quantities, and green denotes product related quantities. Dashed line represents putative causal relation not documented.

Figure 13 depicts QR model M3 (enzyme stalling and surface limitation) as an extension of model M2 encompassing a surface limitation process from the model M1. It accounts for surface crowding by enzyme that, in turn, can hinder the hydrolytic activity (assertion 2). In M1 *Accessible surface* affects the adsorption of enzyme. In model M3, we assume that *Accessible surface* affects the *Adsorption/Complexation rate* of model M2. This way, M3 integrates both the limitation of the adsorption in agreement with M1 (Fig. 8) and the limitation of the complexation (assertion 1). The state of the enzyme covering the cellulose surface is unclear, it is generally suspected that it is adsorbed enzyme as in M1. With the model M2 logic it seems more natural to extrapolate the impact of *Stalled enzyme* at the surface and assume a dependence (P+) between the *Stalled enzyme* and the *Covered surface*. In doing so, we test a mechanism by which *Stalled enzyme* hinders the adsorption and/or the complexation rate. Naturally other hypotheses of this kind could be tested as well, a more complete screening of the possible model structures is actually envisaged in our future work.

M2 and M3 exhibit comparable behaviour. M3 state-graph (see Supplementary Fig. S4) is larger (41 states against 27) and includes more diverse dynamics. M2 and M3 both envision the accumulation of *Stalled enzyme* governed by the balance between the *Stalling rate* and *Desorption rate2*. Inclusion of *Accessible surface* in M3 implements a negative feedback from *Stalled enzyme* concentration to *Adsorption/Complexation rate*. This leads to more complicated oscillations than for model M2 and might reflect a longer establishment of the steady state.

### Validate QR model M3 for theory (encompassment test)

M3 is mainly an augmentation of model M2, therefore it should encompass model M2 publications. Thus, M3 also fulfils the encompassment of the Cruys-Bagger *et al*.^20^ results as it produces the same BP [1 → 2 → 3 → 5 → 6 → 4] as shown in Fig. 11. So, addition of accessible surface model fragment in M3 does not corrupt results obtained with M2.

### Validate QR model M3 for target behaviour (sufficiency test)

Like M2, M3 also meets TB1. Regarding TB2 and TB2′, first steps of the simulation are given in Fig. 12a,c. It shows a restart of hydrolysis in the path [1→2→3] (Fig. 12a) similar to that obtained with M2. From state 3 onward, some BPs are still similar to M2, like [1→2→3→(4,5)] and match TB2 while others diverge from M2 and actually comply with TB2′. One of them starts with [1→2→3→6]. For this path, *Free enzyme* = <Plus, ▲> all along, meaning that fresh enzyme is still being added to the system. However, in state 6 both *Adsorption/Complexation rate* and *Catalytic rate* stabilize (<Plus, ●>), in agreement with stage (t_2_) of TB2′. This early stabilization of the catalytic rate in state 6 can be traced back to *Accessible surface* = <Plus, ▼>, counterbalancing the augmenting *Free enzyme* = <Plus, ▲>, as can be understood from the model structure (Fig. 13). Last stage of TB2′, t_3_, that depicts stabilization of *Free enzyme* is met in the succeeding states (not shown). This simulation shows that surface limitation due to accumulation of inactive enzymes can potentially explain a disrupted restart phenomenon depicted in the Target Behaviour, TB2′ (Table 4). More precisely this assumes a less efficient turnover of the cellobiohydrolases of the system.

## Discussion

In this paper, we have introduced a method to exploit information from the literature to (*i*) assess the explanatory validity of published kinetic model structures by means of qualitative modelling (QR) with respect to observations reported the literature, and (*ii*) to guide conceptualization of new kinetic models. A proof of concept has been developed using two kinetic models addressing enzymatic hydrolysis of cellulose. Analysing qualitative versions of these two models allowed us to identify and clarify discrepancies concerning their simulation and explanation scope. Finally, an augmented QR model with an improved explanation scope has been developed.

Introducing the notion of Target Behaviour (TB) allowed us to define an explicit *interpretation framework* for theories about cellulose hydrolysis, based on experimental findings and observations reported in the literature. The design of TB1, TB2 and TB2′ was developed in interaction with experts, and as such reflects a specific understanding of the results. Designing TBs should thus be seen as a commitment that can be discussed, challenged, updated and validated by the community. While TB1 and TB2 are rather faithful mappings of published observations, TB2′ assumes that some process can counteract the action of newly added enzyme. There is evidence reported in the literature that points in this direction, particularly the restart experiments using cellulose surface cleaning^34,35^. However, the real impact of the cleaning treatment is difficult to ascertain (removal of reaction products limiting possible inhibition, loose of smallest cellulose particles, decreasing of enzyme-substrate complexes). Further examining TB2′ with dedicated experiments is expected to increase the confidence and details of this interpretation. Except for the hydrolysis restart, TB2, our TB proved to be selective for the two models representing kernel theories (“surface limitation” and “stalling”). However it can be argued that both models can be augmented or modified to meet the whole TB. For instance, the maximum surface area is constant in the model M1; if this quantity were to decrease along with the depletion of the substrate, it would cause a slowing-down of the hydrolysis. Therefore building a larger and more diverse TB will naturally improve the interpretation framework, that we have sketched in this work, and make the model augmentation step more conclusive. In this paper, the TB tests the occurrence of 3 qualitative behaviours (Tables 2, 3, 4), hence similar qualitative behaviours occurring at different timescales cannot be distinguished. For instance, the cellulose hydrolysis rate declines over two different timescales (seconds or minutes versus hours or days), and both are caused by different processes. Hence, reaction (3) can include a slow accumulation of denatured enzyme molecules over time to account for a slower and constant decline of the hydrolysis rate^19^. An immediate perspective of this work would be to enrich the modelling system so as to distinguish the timescales of the simulated Behaviour Paths. An interesting solution in this regard, is proposed by Rickel and Porter^23^. Their approach uses indications of time scales of the direct influences (I+ and I−) to select candidate influence graphs susceptible to account for a query about the system behaviour.

Evaluation of models in biochemistry and in bioengineering fields is largely based on the “goodness of fit” of simulation results against experimental data, as well as evaluation of the predictive capacity. This is generally acknowledged as model validation. A model failing this step must have a flaw somewhere, but high goodness of fit does *not necessary* imply global relevance of the model structure and its underlying theory^2,8^. Evaluation of model structure refers to the capacity of a model to provide a *plausible mechanistic explanation* of observations originating from natural phenomenon. For the sake of simplicity, we call this “conceptual validation”. One can find in the literature two main strategies to perform conceptual validation: (*i*) comparison between simulation results and a large diversity of observed behaviours^2^, put forward by Zhang and Lynd^5^ for the hydrolysis of cellulose, and (*ii*) comparison to other models^8^. The former derives from the common belief that the viability of a model structure increases with the number and diversity of the observations it can explain^2^. The latter has been more often addressed in the domain via comparative analysis of concurrent kinetics models of (ligno)cellulose hydrolysis^19,38–40^. Comparative analysis helps pointing out processes that can best account for the observed kinetics, e.g. testing enzyme denaturation, (ir-)reversible inhibition, and so on. Our main contribution is a method that combines the two points. Because the richest source of observations is the literature, which exceeds by far lab-scale experiments, we introduced the concept of TB to integrate observational data from different sources (literature and experiments), as a series of qualitative events (without numerical information). This high-level representation deliberately conserves *only* the salient traits compatible with the majority of experimental results. A TB then provides the reference to compare concurrent qualitative models. Our method delivers validation features which are not available from numerical and mathematical approaches.

Developing and validating quantitative models are important, especially ordinary or partial differential equations have undisputed assets in term of physical interpretation, simulation accuracy and prediction capacity. But, paradoxically the need for accurate numerical information makes conceptual validation more difficult, starting from the difficulty to interpret the goodness of fit with regard to the model structure. Number of parameters of the model, parameterization technique and specific mathematical formulation (e.g. linear *vs* quadratic relation) affect significantly the model outputs, while not being strictly part of the model structure. Thus, comparative analysis studies can report concurrent kinetic models of (ligno)cellulose hydrolysis with equally high goodness of fit^38,40^, which is just another illustration of the non-uniqueness of model results and of the difficulty to assess the “veracity” of a mathematical model in natural science^2^. Secondly, experimental data are often context-dependent, which has two consequences: it makes the parameterized model also context-dependent and it hinders the use of datasets from different sources.

QR modelling circumvents the limitations described above because of two main reasons. Firstly, it captures the model structure as computable cause-effect relations. Consequently, simulation results are directly attributable to this model structure. Moreover, QR simulation generates traces about the system condition at each state, e.g. causal relations at work in a given state and possible transitions^25^. Traces are important information to assess the conceptual relevance of a model^8^. Secondly, being non-parameterized and knowledge-based, QR models are more readily developed and analysed in the context of published documents without the need for data or calibration.

## Conclusion

This work falls within an approach to capture elements of scientific publications as high-level computer models, to address the information overload phenomenon in natural science. A method is presented to assess conceptual validity of kinetic models of cellulose hydrolysis using literature-based information and to support model augmentation. The method is based on two main ideas: the mapping of scientific models using a unique high-level modelling formalism, known as Qualitative Reasoning (QR), and the design of Target Behaviours (TB) from a set of publications, to serve as a reference for model evaluation. The method assesses specifically causal explanations conveyed by scientific models to determine the plausible theories with regard to existing observations. Model augmentation should also lead to a more systematic exploration of the system mechanisms. Many kinetic models of cellulose hydrolysis limitation have been published so far, which makes this topic particularly interesting to further develop and test using such a modelling approach.

As a proof of concept, the new method is applied to assess two published kinetic models of cellulose hydrolysis, with different rate-limitation mechanisms. Three TBs are proposed, which include the hydrolysis rate decline, the restart of hydrolysis due to a second dose of enzyme, and a disrupted restart of hydrolysis. It turned out that none of the kinetic models can account for all the aspects defined by the TBs. For this reason, a third model was developed using elements from of the preceding models. The design of the third model illustrates how the proposed method can foster the formulation and testing of new paradigms with a larger explanatory scope regarding information reported in the literature.

## Electronic supplementary material

Supplementary Information

## References

[1] Fraser, A. G. & Dunstan, F. D. On the impossibility of being expert. BMJ 341 (2010). 10.1136/bmj.c6815 21156739

[2] Oreskes N, Shrader-Frechette K, Belitz K (1994). Verification, Validation, and Confirmation of Numerical Models in the Earth. Sciences. Science.

[3] Zhang YH, Lynd LR (2004). Toward an aggregated understanding of enzymatic hydrolysis of cellulose: noncomplexed cellulase systems. Biotechnol Bioeng.

[4] Bansal P, Hall M, Realff MJ, Lee JH, Bommarius AS (2009). Modeling cellulase kinetics on lignocellulosic substrates. Biotechnology Advances.

[5] Zhang YH, Lynd LR (2006). A functionally based model for hydrolysis of cellulose by fungal cellulase. Biotechnol Bioeng.

[6] Forbus, K. D. Qualitative Modeling in Handbook of knowledge representation, edited by Vladimir Lifschitz Frank van Harmelen, Bruce Porter (Elsevier, New York, Vol. Volume 3, pp. 361–394, 2008).

[7] Travé-Massuyès L, Ironi L, Dague P (2004). Mathematical foundations of qualitative reasoning. AI Mag..

[8] Rykiel EJ (1996). Testing ecological models: the meaning of validation. Ecological Modelling.

[9] Lynd, L. R., Weimer, P. J., van Zyl, W. H., & Pretorius, I. S. Microbial cellulose utilization: fundamentals and biotechnology. Microbiol *Mol Biol Rev* **66**(3), 506–577, table of contents (2002). 10.1128/MMBR.66.3.506-577.2002 PMC120791 12209002

[10] Väljamäe P, Kipper K, Pettersson G, Johansson G (2003). Synergistic cellulose hydrolysis can be described in terms of fractal-like kinetics. Biotechnology and Bioengineering.

[11] Kafle K, Shin H, Lee CM, Park S, Kim SH (2015). Progressive structural changes of Avicel, bleached softwood, and bacterial cellulose during enzymatic hydrolysis. Sci Rep.

[12] Hu J (2015). The accessible cellulose surface influences cellulase synergism during the hydrolysis of lignocellulosic substrates. ChemSusChem.

[13] Linder M, Teeri TT (1997). The roles and function of cellulose-binding domains. Journal of Biotechnology.

[14] Valjamae P, Sild V, Pettersson G, Johansson G (1998). The initial kinetics of hydrolysis by cellobiohydrolases I and II is consistent with a cellulose surface-erosion model. Eur J Biochem.

[15] Sugimoto N, Igarashi K, Wada M, Samejima M (2012). Adsorption characteristics of fungal family 1 cellulose-binding domain from Trichoderma reesei cellobiohydrolase I on crystalline cellulose: negative cooperative adsorption via a steric exclusion effect. Langmuir.

[16] Maurer SA, Bedbrook CN, Radke CJ (2012). Cellulase Adsorption and Reactivity on a Cellulose Surface from Flow Ellipsometry. Industrial & Engineering Chemistry Research.

[17] Eriksson T, Karlsson J, Tjerneld F (2002). A model explaining declining rate in hydrolysis of lignocellulose substrates with cellobiohydrolase I (cel7A) and endoglucanase I (cel7B) of Trichoderma reesei. Appl Biochem Biotechnol.

[18] Jalak J, Valjamae P (2010). Mechanism of initial rapid rate retardation in cellobiohydrolase catalyzed cellulose hydrolysis. Biotechnol Bioeng.

[19] Praestgaard E (2011). A kinetic model for the burst phase of processive cellulases. FEBS J.

[20] Cruys-Bagger N (2012). Pre-steady-state Kinetics for Hydrolysis of Insoluble Cellulose by Cellobiohydrolase Cel7A. J. Biol. Chem..

[21] Shang BZ, Chu J-W (2014). Kinetic Modeling at Single-Molecule Resolution Elucidates the Mechanisms of Cellulase Synergy. ACS Catalysis.

[22] Igarashi K (2011). Traffic jams reduce hydrolytic efficiency of cellulase on cellulose surface. Science.

[23] Rickel, J. and Porter, B. Automated modeling of complex systems to answer prediction questions. Artificial Intelligence **93**(1), p. 201–260, 1997).

[24] Bredeweg B, Forbus K (2004). Qualitative modeling in education. AI Mag..

[25] Bredeweg B, Linnebank F, Bouwer A, Liem J (2009). Garp3 - Workbench for qualitative modelling and simulation. Ecological Informatics.

[26] Bredeweg B, Salles P, Nuttle T (2007). Using exogenous quantities in qualitative models about environmental sustainability. AI Commun..

[27] Falkenhainer B, Forbus KD (1991). Compositional modeling: finding the right model for the job. Artif. Intell..

[28] Kuipers, B. Qualitative reasoning: modeling and simulation with incomplete knowledge (MIT press, 1994).

[29] Kansou K, Bredeweg B (2014). Hypothesis assessment with qualitative reasoning: Modelling the Fontestorbes fountain. Ecological Informatics.

[30] Dondelinger E (2016). Contrasted enzymatic cocktails reveal the importance of cellulases and hemicellulases activity ratios for the hydrolysis of cellulose in presence of xylans. AMB Express.

[31] Nidetzky B, Zachariae W, Gercken Gn, Hayn M, Steiner W (1994). Hydrolysis of cellooligosaccharides by Trichoderma reesei cellobiohydrolases: Experimental data and kinetic modeling. Enzyme and Microbial Technology.

[32] Hong J, Ye X, Zhang YHP (2007). Quantitative Determination of Cellulose Accessibility to Cellulase Based on Adsorption of a Nonhydrolytic Fusion Protein Containing CBM and GFP with Its Applications. Langmuir.

[33] Gan Q, Allen SJ, Taylor G (2003). Kinetic dynamics in heterogeneous enzymatic hydrolysis of cellulose: an overview, an experimental study and mathematical modelling. Process Biochemistry.

[34] Bommarius AS (2008). Cellulase kinetics as a function of cellulose pretreatment. Metabolic Engineering.

[35] Yu Z, Jameel H, Chang H-M, Philips R, Park S (2012). Evaluation of the factors affecting avicel reactivity using multi-stage enzymatic hydrolysis. Biotechnology and Bioengineering.

[36] Yang B, Willies DM, Wyman CE (2006). Changes in the enzymatic hydrolysis rate of Avicel cellulose with conversion. Biotechnology and Bioengineering.

[37] Bansal P (2012). Elucidation of cellulose accessibility, hydrolysability and reactivity as the major limitations in the enzymatic hydrolysis of cellulose. Bioresource Technology.

[38] Bezerra RM, Dias AA (2004). Discrimination among eight modified michaelis-menten kinetics models of cellulose hydrolysis with a large range of substrate/enzyme ratios: inhibition by cellobiose. Appl Biochem Biotechnol.

[39] Brown RF, Agbogbo FK, Holtzapple MT (2010). Comparison of mechanistic models in the initial rate enzymatic hydrolysis of AFEX-treated wheat straw. Biotechnol Biofuels.

[40] Pihlajaniemi V, Sipponen MH, Kallioinen A, Nyyssola A, Laakso S (2016). Rate-constraining changes in surface properties, porosity and hydrolysis kinetics of lignocellulose in the course of enzymatic saccharification. Biotechnol Biofuels.

