# Supplementary material for: Testing scientific models using Qualitative Reasoning: Application to cellulose hydrolysis
Source: Sci Rep. 2017 Oct 26;7:14122. doi: 10.1038/s41598-017-14281-4 (PMC5658447; doi:10.1038/s41598-017-14281-4)
Supplement: Supplementary file 1 — Supplementary Information [file 41598_2017_14281_MOESM1_ESM.pdf]

Supplementary Information

For "Testing scientific models using Qualitative Reasoning: Application to cellulose hydrolysis"

Kamal Kansou[1*], Caroline Rémond[2], Gabriel Paës[2], Estelle Bonnin[1], Jean Tayeb[2], Bert Bredeweg[3]

[1]INRA, Biopolymères Interactions et Assemblages, BP 71267, 44316 Nantes, France
[2]FARE laboratory, INRA, University of Reims Champagne-Ardenne, 51100 Reims, France
[3]Informatics Institute, University of Amsterdam, Science Park 904, 1098 XH Amsterdam, The Netherlands

*To whom correspondence should be addressed. Tel: +33 (0)2 40 67 51 49, Email: kamal.kansou@inra.fr

APPENDIX

Model M1 encompassment test information

Reduced amount of Free enzyme. Figure S1 simulation curves are produced from a model built after equations given in Maurer et al.[1], with inputs describing a reduced amount of free enzyme compared to the available surface. In Figure S1, one can notice the consecutive monotonic increase of the amount of adsorbed enzyme (red curve) and then amount of complexed enzyme (blue curve), prior to stabilization. Neither the adsorption rate nor the catalytic rate decrease. Figure S1 depicts a limitative absorption rate compared to complexation and desorption rates. QR Model M1, produces the pathway [1→2→3→4→5] (Fig. 10ab), which encompasses this behaviour.

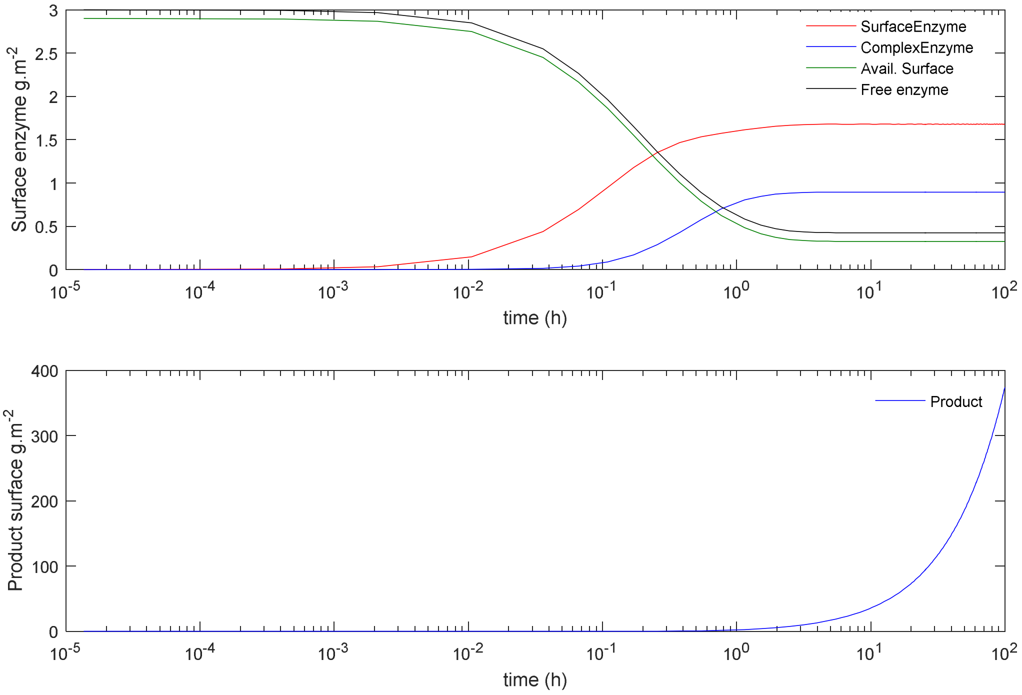

Figure S1. Simulation from kinetic model described in Maurer et al.,[1], with limitative amount of Free enzyme for 100h hydrolysis

Excess of Free enzyme. Figure S2 simulation curves are produced with an excess of Free enzyme compared to the available surface. The amount of Free enzyme is too high to be shown on the graph. One can notice the peak of adsorbed surface enzyme (red curve), which denotes a bottleneck effect at the adsorption stage due to limitative complexation rate. QR Model M1 produces the pathway [1→2→3→4→6→7→5] (Fig. 10ab), which encompasses this behaviour.

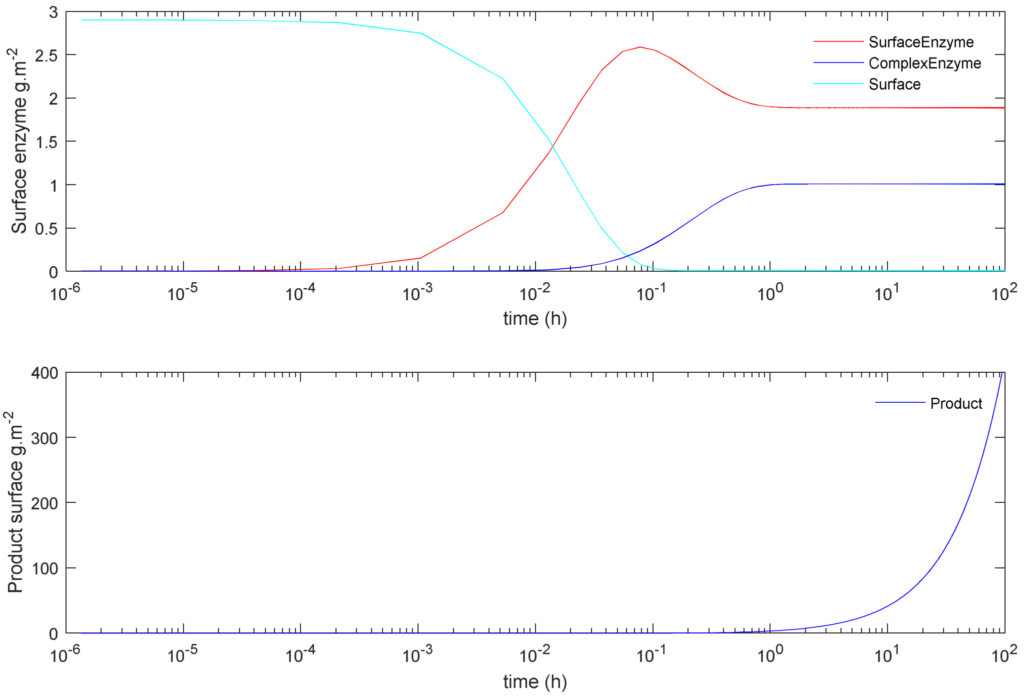

Figure S2. Simulation from kinetic model described in Maurer et al.[1], with excess of Free enzyme for 100h hydrolysis. One can notice the peak of adsorbed enzyme.

Model M1, sufficiency test information

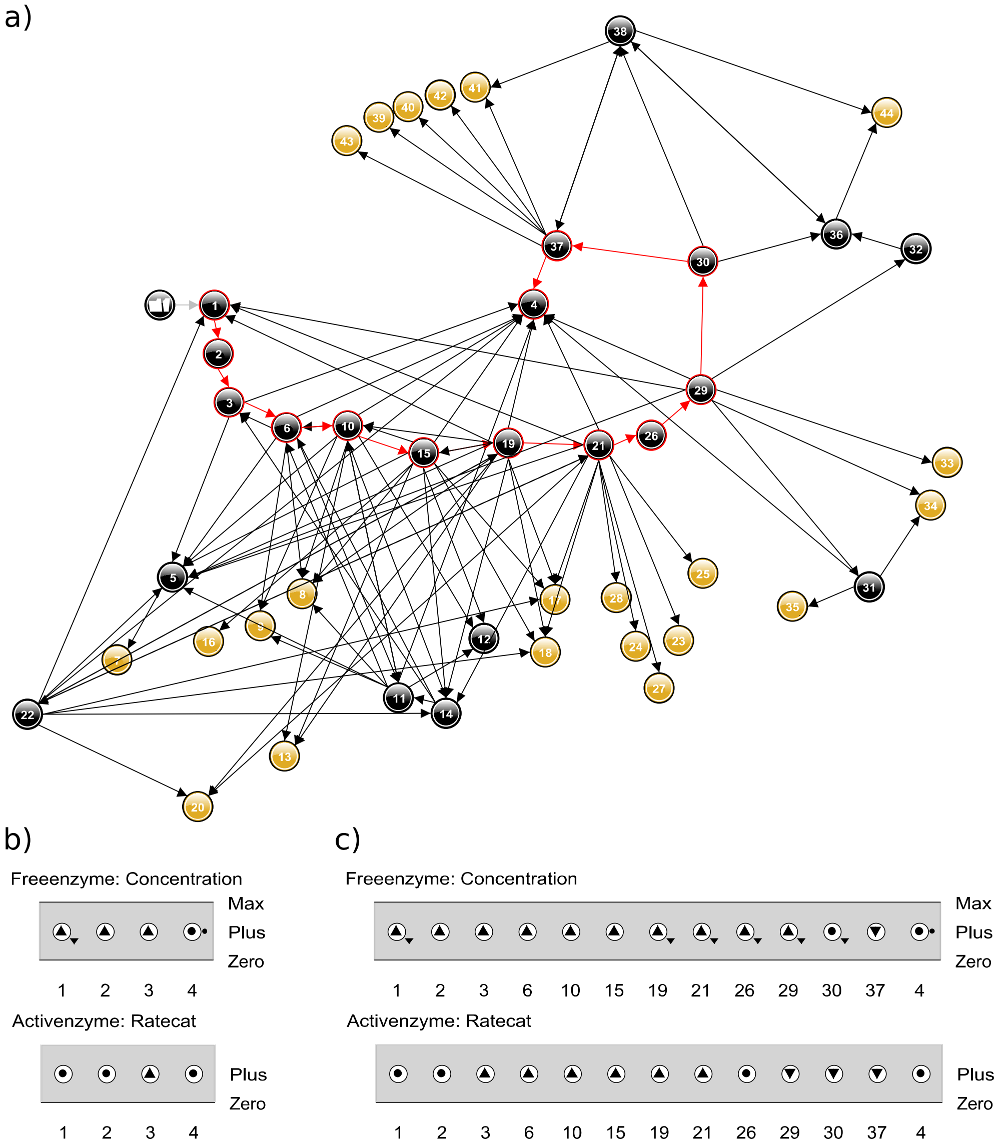

Figure S3. Results for M1 sufficiency test for TB2 and TB2' (restart scenario). Partial state-graph of model M1 (a). Value histories of selected quantities depicting the behaviour path [1→2→3→4] consistent with TB2 (Tab. 3) (b). Value histories of selected quantities depicting the behaviour path [1→2→3→6→10→15→19→21→26→29→30→37→4] consistent with TB2' (Tab. 4) (c).

Model M3, simulation results

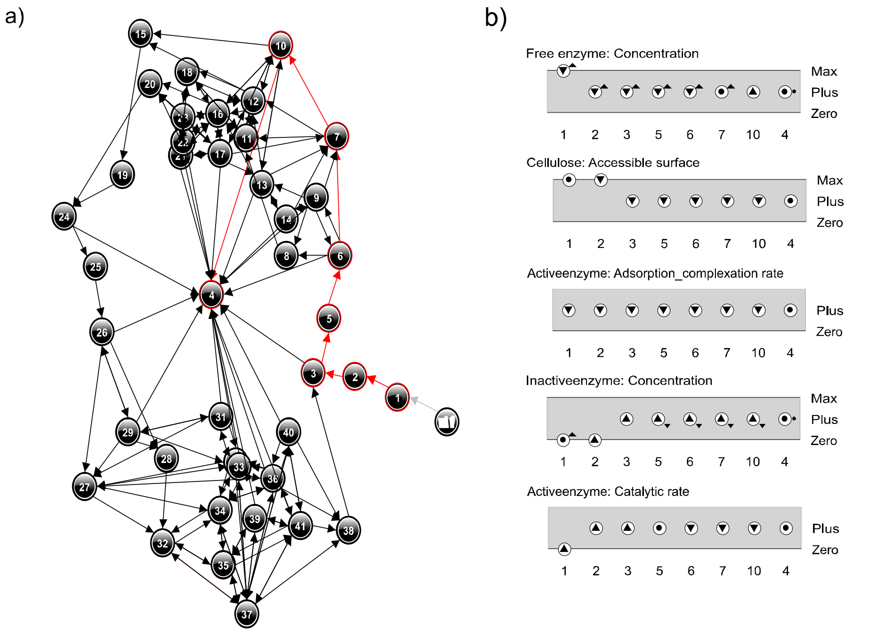

Figure S4. Simulation results for M3. State-graph of model M3 of 41 distinct states with one end-state (state 4) (a). Value histories of selected quantities for model M3 depicting the behaviour path [1→2→3→5→6→10→4] (b).

References

1 Maurer, S.A., Bedbrook, C.N., & Radke, C.J., Cellulase Adsorption and Reactivity on a Cellulose Surface from Flow Ellipsometry. Industrial & Engineering Chemistry Research 51 (35), 11389-11400 (2012).
